# Best practices for instrumenting honey bees

**DOI:** 10.1038/s41598-022-16168-5

**Published:** 2022-07-27

**Authors:** Phoebe A. Koenig, Kirstin H. Petersen

**Affiliations:** 1 Department of Entomology, Cornell University, Ithaca, NY 14850 USA; 2 Department of Electrical and Computer Engineering, Cornell University, Ithaca, NY 14850 USA

**Keywords:** Ecology, Behavioural ecology, Conservation biology, Ecosystem services, Electrical and electronic engineering

## Abstract

Honey bees are vital pollinators and can be used to monitor the landscape. Consequently, interest in mounting technologies onto bees to track foraging behaviors is increasing. The barrier to entry is steep, in part because the methodology for fastening tags to bees, and the success rates, are often missing from publications. We tested six factors suspected to influence the presence and tag retention rates of nurse honey bees after their introduction to hives, and followed bees until foraging age. We also compared reintroducing foragers to their maternal colony using the best method for nurse bees to releasing them in front of their maternal hive and allowing them to fly back unaided. Nurses were most likely to be present in the hive with their tag still attached when introduced using an introduction cage at night. Glue type was important, but may further be influenced by tag material. Foragers were most likely to be present with a tag attached if released in front of their colony. Preparation and introduction techniques influence the likelihood of tagged honey bee survival and of the tags remaining attached, which should be considered when executing honey bee tagging and tracking experiments.

## Introduction

Researchers have been marking and tagging honey bees for over 70 years^1^. Individualized tags allow us to learn how behaviors change with age, interventions, and environmental context^2–6^. In addition to informing natural history, honey bee tracking studies can be used to identify and monitor environmental contaminants, detect explosives, monitor agricultural blooms, and track pollination activity^7–11^.

Previously, foraging honey bees have been tracked with external sensors like LIDAR and harmonic radar^12,13^. With the miniaturization of electronics, engineers are integrating tracking functionality directly into bee tags. One way is to use radio-frequency identification (RFID) technology, however, this method is limiting because bees with RFID tags can only be tracked when they are near a reader, making it necessary to scatter readers throughout the environment in order to reconstruct bee flights^14,15^. More recently, researchers have shown that it’s possible to design custom chips, or Application Specific Integrated Circuits (ASICs), to record information about bee movement continuously throughout a foraging flight^16,17^. Successful design and execution of honey bee tracking with ASICs may provide valuable insight into landscape health, the spread of environmental contaminants, and agricultural management.

However, honey bees live in social colonies, which can pose difficulties to successful tagging. They readily groom each other and can identify and reject non-kin intruders^18,19^. Rejection and tag removal both prevent data collection. The full methodology for tagging honey bees and introducing them to a colony, and the resulting success rates, are rarely reported in biology papers. Consequently, the barrier to enter this emerging, interdisciplinary field is high; it can be prohibitively difficult to mount tags on live bees, let alone successfully introduce tagged bees to honey bee colonies and have the tags remain on the bees long enough to harvest useful data. Since the foraging patterns of honey bees can give us valuable insight into landscape health, the location of environmental contaminants, and agricultural management, it is in our best interest to reduce the barrier of entry and encourage more engineers to develop tools to track honey bees. Inspired by honey bee natural history and current practices in bee labs, we tested techniques that could influence the acceptance rate of tagged bees into a hive and the tag retention rates. Additionally, we considered the type of tag, material accessibility, and labor requirements, as these factors affect turnaround times.

## Methods

### Experiment 1

**Figure 1:**
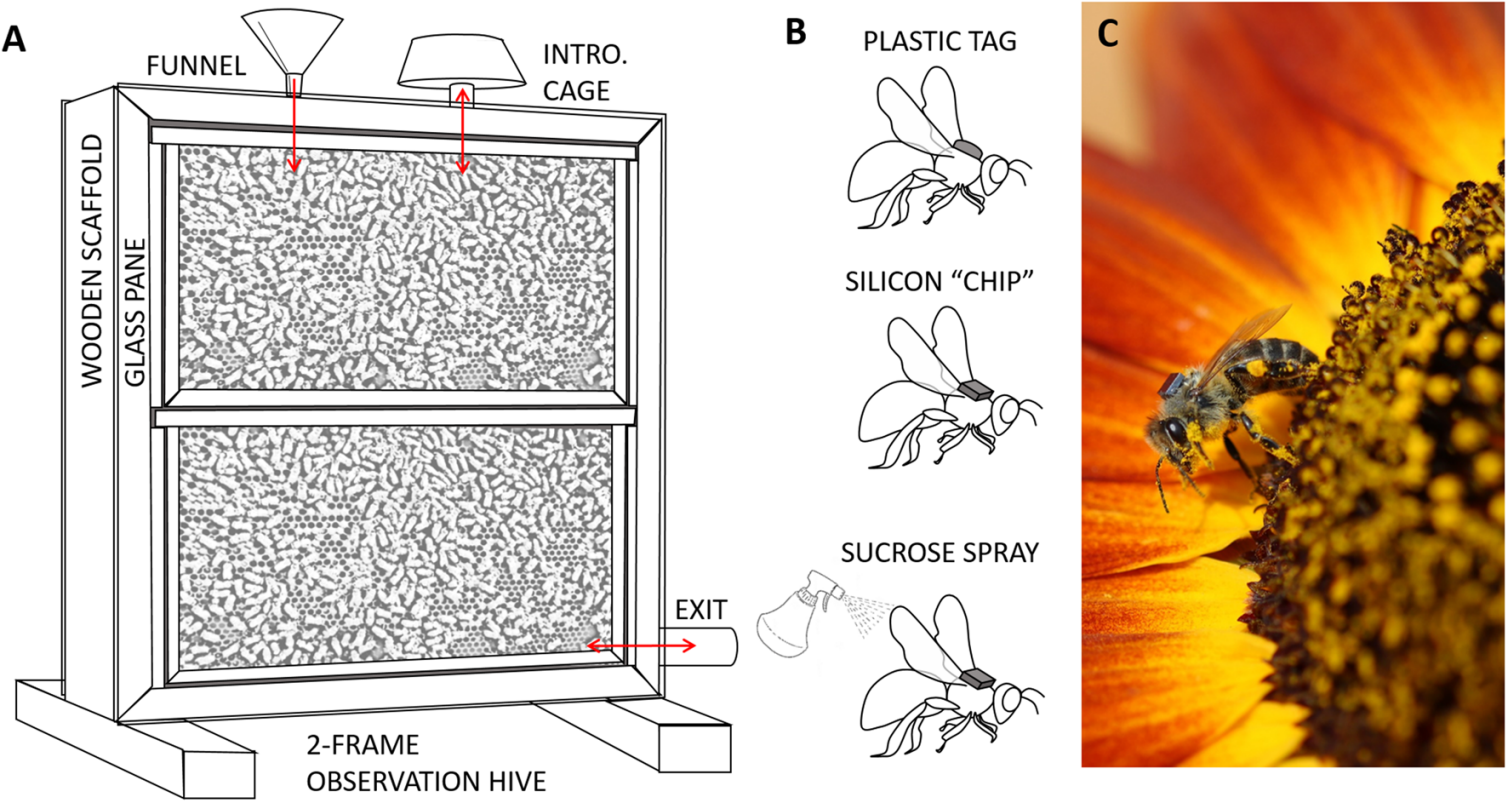
(**A**) Observation hive with introduction holes (red), through which bees were introduced via funnel or introduction cage. (**B**) Plastic tags, silicon tags, and sucrose spray. (**C**) Photograph of a tagged bee foraging (Photo by Greg Yauney).

To study the acceptance and tag retention rates of honey bees under different introduction conditions, we set up three two-frame observation hives with $\sim 1500$ adult bees and a queen. Observation hives were set up in a shed, with an entrance tube that connected to the outside 4 ft above the ground so bees could freely forage in the surrounding fields (Fig. 1A). We put emerging brood from healthy source colonies in an incubator ($33.5^{\circ }$ C, $\ge 55$% RH) and tagged individuals that emerged overnight. Each hive had two vent holes (1” Dia.) through which we could introduce bees (Fig. 1A).

In our initial experiment, there were seven treatment groups with 20 bees each per colony (n = 420 bees total). The seven treatments were: Control (C), No Sucrose (NS), Plastic (P), Wood Glue (WG), Not Incubated (NI), No Cage (NC), and Day (D). The Control treatment was designed to be a positive control, where we applied all the techniques we thought might increase acceptance of bees into a colony and tag retention rates. All other treatments had a single difference in the tag, tagging process, or introduction process to distinguish it from the control group, as detailed in Table 1. We glued a tag to the thorax of each bee (Fig. 1B) and marked the abdomen with a paint pen (Posca) to distinguish among treatment groups. In order to glue tags on, we picked each bee up, placed a small amount of glue on the thorax, and placed a tag on top of the glue with a pair of forceps (see Video 1 which details the tagging process). All bees except those in the Plastic group were tagged with 1.7 mm$^2$ silicon tags (3.4 mm area). Silicon was chosen because it is a material representative of ASICs, which you would expect in a custom chip designed to track bee foraging flights. Plastic tags were 3 mm Dia. plastic discs (7.07 mm area), which are the commercially available bee tags commonly used in honey bee tracking and behavior experiments (Betterbee). All tags were glued on with shellac glue, the glue that comes with commerical honey bee marking kits (Betterbee), except for in the wood glue group, where they were glued on with wood glue (Titebond III). Next, bees were placed in a container with a bit of honey and stored until they were ready to be introduced. All bees except those in the Not Incubated group were placed in the incubator ($33.5^{\circ }$ C, $\ge 55$% RH). The Not Incubated group was stored in a room environment, with variation between 21–27$^{\circ }$ C and 35–42% RH until introduction. Bees in the Day treatment spent 5 h in the incubator and then were sprayed with a light sucrose syrup (1 sucrose: 1 water (v/v)) and introduced at 4pm while the hives were still actively foraging. The rest of the bees spent between 5 and 8 h in the incubator or room environment before being introduced at 10:30 pm, after foraging had concluded. All except the No Sucrose group were sprayed with a light sucrose syrup before being introduced. The No Cage bees were rapidly introduced through one of the vent holes on the top of the hive using a funnel. The rest of the bees were placed in a cage together, which we connected to the introduction holes at the top of the colony, allowing them to move freely between the cage and the hive.

**Table 1:** Experimental design used for preparation and introduction of treatment groups.

Treatment	Sucrose	Tag	Glue	Incubator	Intro method	Intro time	Bee type	n$^1$
**Experiment 1**								
Control (C)	Y	Silicon	Shellac	Y	Cage	Night	Nurse	60
No Sucrose (NS)	$\mathbf{N}$	Silicon	Shellac	Y	Cage	Night	Nurse	60
Plastic (P)	Y	**Plastic**	Shellac	Y	Cage	Night	Nurse	60
Wood Glue (WG)	Y	Silicon	**Wood**	Y	Cage	Night	Nurse	60
Not Incubated (NI)	Y	Silicon	Shellac	**N**	Cage	Night	Nurse	60
No Cage (NC)	Y	Silicon	Shellac	Y	**Funnel**	Night	Nurse	60
Day (D)	Y	Silicon	Shellac	Y	Cage	**Day**	Nurse	60
**Experiment 2**								
Wood Glue 2 (WG2)	Y	Silicon	**Wood**	Y	Cage	Night	Nurse	58
Superglue (SG)	Y	Silicon	**Super**	Y	Cage	Night	Nurse	59
**Experiment 3**								
Hive Introduced (HI)	Y	Silicon	Wood	Y	Cage	Night	**Forager**	60
Natural Release (NR)	Y	Silicon	Wood	Y	**Outside**	**Day**	**Forager**	60

$^1$ n denotes number of bees per treatment (20 per colony, 60 total). Some died before introduction

Parameters that differ from the control are in [bold].

Beginning on day 2 (07/09/2020), we observed each hive in the morning on days 2-4 and 6-9 to see how many bees per group were present, hereinafter referred to as presence, and how many bees per group were present with tags, hereinafter referred to as success. We selected a random order in which to observe the three hives and a random order in which to observe the treatment groups for each hive. Each side of each hive had a grid drawn on it that divided it into nine squares. We scanned each side of each colony by eye for each treatment, starting with the lower left square of the grid on the first side, moving across the row, and then moving up to the next row, counting presence and success, using a tally counter when needed. We then moved rapidly to the other side and started at the top left of the grid, scanning row by row until we had observed each square in the grid. After an initial scan for each treatment, we placed the covers on the hives and shook for 10s to encourage bees to move around in the hive, and then waited for at least 15 minutes before a second observation. The maximum presence and success from the two daily observations were used for each treatment group and hive for analysis. Since we collected data by scanning each colony, we sometimes found more bees from a group in an observation hive than we had found in the same hive on previous day(s), even though more time had passed. Over the course of the experiment, our hives grew in size, and we believed we were seeing less tagged bees in part because they made up a smaller proportion of the hive population, and so decided to do a destructive sampling before the tagged bees reached foraging age. After dark on day 14 (7/21/2020), we made sure no tagged bees were dead on the bottom of the hives. We blocked the entrances, vacuumed all bees at the entrances into containers, and froze vacuumed bees and the three colonies, so that we could do a destructive sampling of all 3 colonies. This allowed us to get a final count of the presence and success for each of the seven treatment groups. We dissected each frozen colony, removing and inspecting each dead bee, and recorded the presence and success of each treatment group.

### Experiments 2 and 3

We set up three two-frame observation hives in the same shed used for experiment 1 to conduct follow-up experiments in August 2020. The goal of experiment 2 was to compare Gorillaglue gel, an easily accessible Superglue (SG), to Titebond III, a readily accessible Wood Glue (WG2) used in experiment 1. We placed frames of capped brood in an incubator overnight to produce one day old nurse bees. We picked up each bee, placed a small dot of either superglue or wood glue on the thorax, and then placed 1.7 mm$^2$ silicon tags on top of the glue. Bees were stored in the incubator with honey for 5–6 h until after dark. Then, we sprayed the bees with a light sucrose syrup and connected their cages to the vent holes at the top of the observation hives, allowing the bees to freely move between their cage and the hives. These details are summarized in Table 1.

Some honey bee tagging projects may benefit from tagging foragers as opposed to nurse bees, because nurse bees are the youngest workers and if you tag them you must wait for them to reach foraging age, during which time they may lose their tags. Specifically, tagging foragers as opposed to nurses will be advantageous when the tag price is extremely high or the project is very time constrained, and knowing the exact age of tagged bees is not important for the project goals. Since foragers are older workers that have already acquired the colony scent and learned to navigate the area surrounding their hive, the optimal methods for introducing nurses and foragers may differ. It is not easy to use bees from a source colony, because if they are within foraging range of their maternal colony, they will attempt to fly back home. The goal of experiment 3 was to apply a treatment that had high success with nurse bees (Experiment 1: WG) to foragers, and compare with releasing foragers near their colony and allowing them to return freely. We call these treatments Hive Introduced (HI) and Natural Release (NR), respectively. All foragers for this experiment were collected from the observation hives and were introduced back to the same observation hive after tagging, either through the vent holes at the top of the hive or by releasing the bees near the entrance of the hive. We collected foragers from each colony entrance into a cage with an insect vacuum (Hand-Held DC Vac/Aspirator, Bioquip), specifically aspirating bees that were arriving from foraging trips or had nectar loads, and placed them in the fridge to anesthetize them. We then selected those with intact wings, placed a dot of wood glue on their thoraxes, and placed silicon tags on top of the glue. Both treatment groups were stored in the incubator ($33.5^{\circ }$ C, $\ge 55$% RH) and given honey to feed on. After 2 h in the incubator, the containers with NR bees were sprayed with a light sucrose syrup and placed on the ground 5 ft in front of their respective hive entrances and opened, allowing the bees to fly back to their hives unaided. At 10PM, when it was dark and foraging had concluded, the HI bees were sprayed with a light sucrose syrup. Their cages were then connected to the vent holes at the top of the observation hives, allowing them to freely move between their cage and the hives.

Experiments 2 and 3 were conducted in the same hives simultaneously, but were considered separate experiments because experiment 2 was conducted with nurses of known age and experiment 3 was conducted with foragers of unknown age. Nurses and foragers typically have an age difference and experience different levels of risk due to the behaviors they engage in, and so we analyzed these data separately in order to not confound our results. Beginning on day two (08/26/2020), we observed each hive on days 2–11 and 15–21 to determine introduction presence and success for experiment 2 and experiment 3. Forager observations (experiment 3) were always done early in the morning, before foraging activity commenced. As in experiment 1, we randomized observation order, scanned colonies for each treatment group before and after shaking, and used the maximum presence and success from the two observations for analysis. Since we collected data on multiple days by scanning each colony, we occasionally found more bees in a group than we had found on previous day(s), even though more time had passed.

### Statistical methods

Statistical analyses were performed in R 4.0.5^20^. To determine which preparation and introduction techniques were associated with the highest presence and success, we built generalized linear mixed-effects models (glmms)^21^ for the proportion of present and success bees to introduced bees respectively, with treatment and sampling day as fixed effects, and colony as a random effect. For experiment 1, treatment was a categorical variable, where the Control bees were the reference group. We assessed the significance of the full models using Wald likelihood ratio chi-square tests on each glmm (‘Anova’ function in the ‘car’ package with test set to ‘Chisq’)^22^. In all statistical tests, $\alpha$ was set to 0.05. The destructive data from experiment 1 were analyzed separately from hive observation data. We ran a correlation test to determine the relationship between hive observation data from the final observation day, day 9, and the destructive sampling on day 14 using the ggpubr package^23^.

## Results

**Figure 2:**
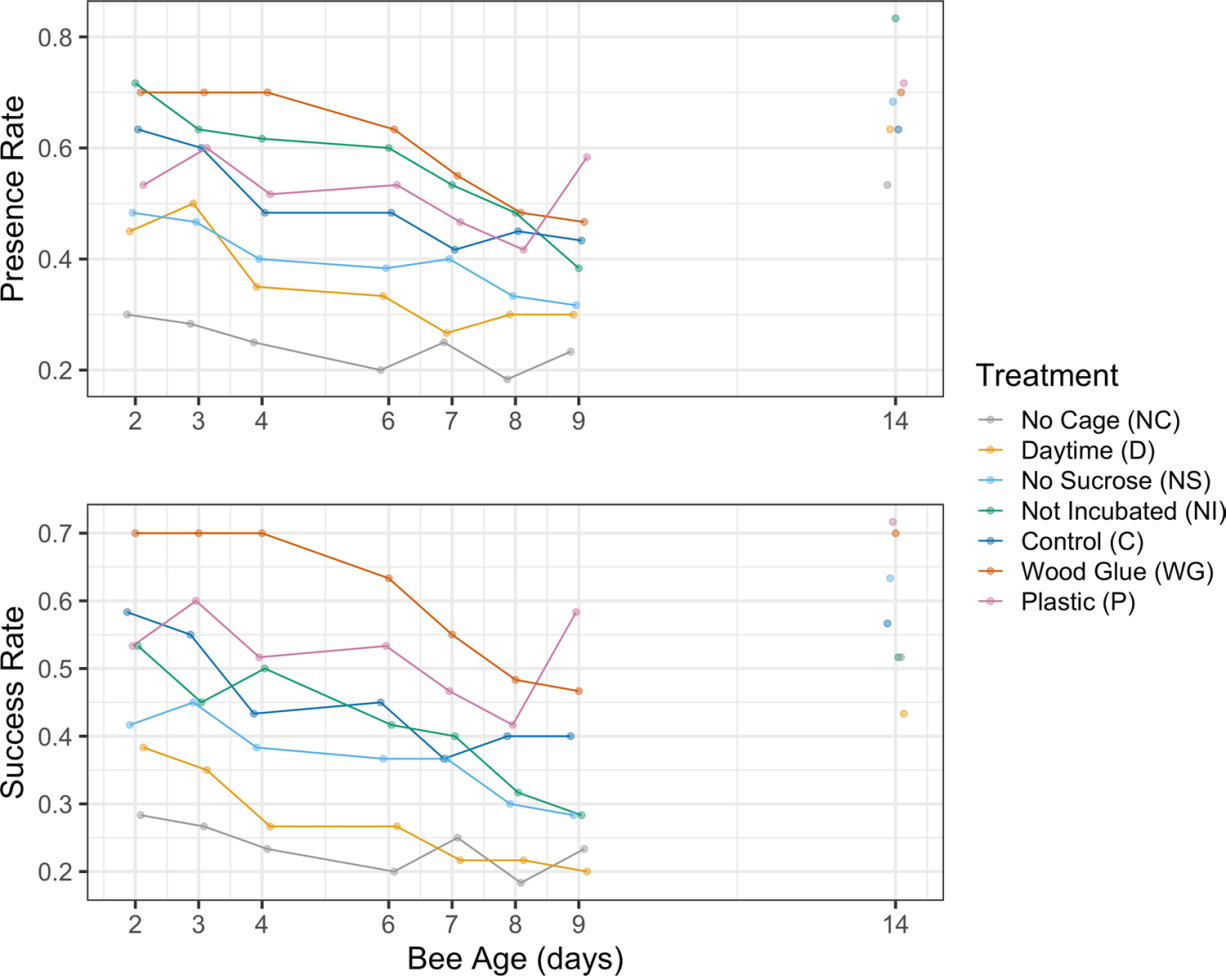
Presence and success for nurse bees in the seven treatment groups on the days following introduction to observation hives. Day 14 results are from the destructive sampling, where each of the three hives were frozen, and all bees inside were inspected for paint and tags.

In experiment 1, treatment group (presence: Wald $$X^2$$, $$X^2=163.8$$, $$p<0.001^{***}$$, success: Wald $$X^2$$, $$X^2=174.5$$, $$p<0.001^{***}$$) and bee age (presence: Wald $$X^2$$, $$X^2=47.4$$, $$p<0.001^{***}$$, success: Wald $$X^2$$, $$X^2=40.2$$, $$p<0.001^{***}$$) significantly affected presence and success. The number of present and success bees significantly decreased as the age of the bees increased (Fig. 2). The Wood Glue treatment had increased presence and success compared to the control, which used shellac glue. The No Cage, No Sucrose, and Day treatment groups all had significantly lower presence and success when compared to the control, which used an introduction cage, sucrose spray, and nighttime introduction. The Not Incubated treatment group, which was kept at room temperature before introduction, had significantly higher presence and slightly, but not significantly, lower success when compared to the control group. The Plastic group had slightly increased presence and success compared to the control, which used silicon tags, though this difference was not statistically significant. Results for each treatment with respect to the control group, which was used as the reference group in our glmms, are in Table 2.

**Table 2:** Glmm results for models analyzing the fixed effect of treatments on presence and success over the course of the hive observations, where treatments are compared to the Control group. Asterisk denotes statistical significance with respect to the control group ($\alpha =0.05$), alongside mean and se of presence and success on day two.

Treatment	Presence			Success		
	Z-value	*p* value	Mean±se	Z-value	*p* value	Mean±se
Wood Glue (WG)	3.09	0.002**	$70\%\pm 3$	4.37	< 0.001***	$70\%\pm 3$
No Cage (NC)	− 7.68	< 0.001***	$30\%\pm 5$	− 6.63	< 0.001***	$28\%\pm 7$
Day (D)	− 4.22	< 0.001***	$45\%\pm 5$	− 5.52	< 0.001***	$38\%\pm 7$
No Sucrose (NS)	− 3.02	0.003**	$48\%\pm 4$	− 2.61	0.009**	$42\%\pm 4$
Not Incubated (NI)	1.96	<0.05*	$72\%\pm 10$	− 1.19	0.23	$53\%\pm 12$
Plastic (P)	0.63	0.53	$53\%\pm 6$	1.95	0.05	$53\%\pm 6$
Control (C)			$63\%\pm 10$			$58\%\pm 7$
Age (Days)	− 6.88	< 0.001***		− 6.34	< 0.001***	

**Figure 3:**
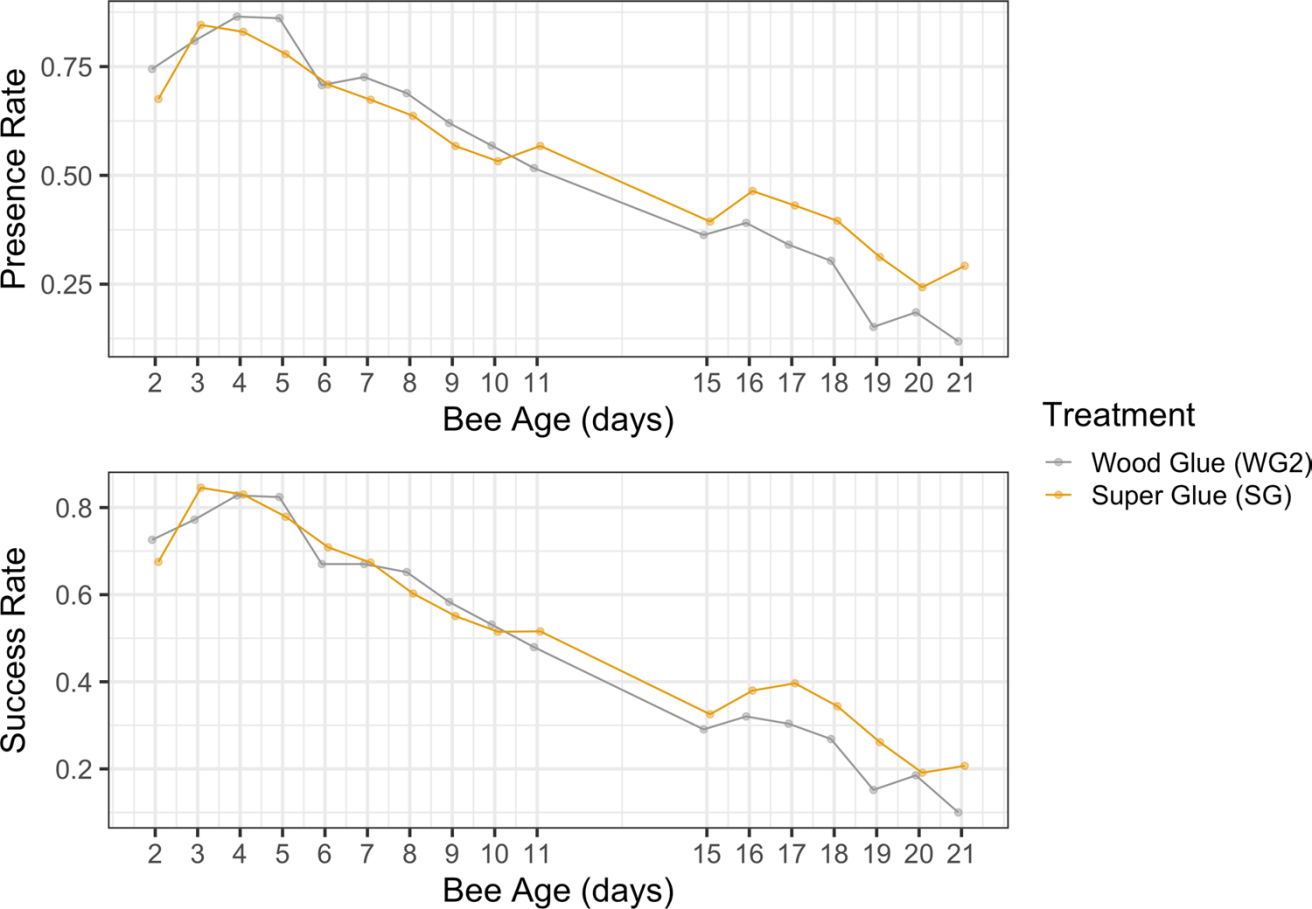
Presence and success of nurses on the days following introduction to the three observation hives. Bees were introduced at 1 day old.

In the destructive sampling, treatment group was, overall, a significant predictor of both presence and success (Presence: Wald $$X^2$$, $$X^2=13.6$$, $$p=0.03^{*}$$, success: $$X^2=15.8$$, $$p=0.01^{*}$$). The Wood Glue, No Sucrose, and Plastic treatments all had slightly increased presence and success when compared to the control, where silicon tags were glued on with shellac and the bees were sprayed with sucrose before introduction, but these differences were not significant. The No Cage and Day treatments both had lower presence and success than the control, which used an introduction cage and nighttime introduction, but these differences were not significant. The Not Incubated treatment had a significantly higher presence than the control, but had slightly, but not significantly, lower success. Results with respect to the control group, which was the reference group in our glmms, are in Table 3. The presence and success rates during hive observations on day 9 were significantly correlated with the results of the destructive sampling on day 14 (Pearson’s Correlation Test, Presence: t = 2.45, $$p=0.02^{*}$$, success: t = 3.5, $$p=0.002^{**}$$).

**Table 3:** Glmm results for models analyzing the fixed effect of treatment on presence and success during the destructive sampling (day 14), where treatments are compared to the Control group. Asterisk denotes statistical significance with respect to the control group ($\alpha =0.05$), alongside mean and se for presence and success.

Treatment	Presence			Success		
	Z-value	*p* value	Mean±se	Z-value	*p* value	Mean±se
Wood Glue (WG)	0.78	0.43	$70\%\pm 10$	1.51	0.13	$70\%\pm 10$
No Cage (NC)	− 1.12	0.26	$53\%\pm 9$	− 0.55	0.58	$52\%\pm 8$
Day (D)	0	1	$63\%\pm 8$	− 1.46	0.15	$43\%\pm 4$
No Sucrose (NS)	0.58	0.56	$68\%\pm 9$	0.75	0.46	$63\%\pm 7$
Not Incubated (NI)	2.46	0.01*	$83\%\pm 10$	− 0.55	0.58	$52\%\pm 7$
Plastic (P)	0.99	0.32	$72\%\pm 2$	1.70	0.09	$72\%\pm 2$
Control (C)			$63\%\pm 6$			$57\%\pm 7$

In experiment 2, treatment group did not significantly affect presence or success (presence: Wald $$X^2$$, $$X^2=1.1$$, $$p=0.30$$, success: Wald $$X^2$$, $$X^2=1.3$$, $$p=0.25$$). Super Glue bees had slightly higher presence and success over the course of the sampling period than Wood Glue bees, but this difference was not significant (presence: glmm, $$z=-1.05$$, $$p=0.29$$, success: glmm, $$z=-1.16$$, $$p=0.25$$). Presence and success for SG were both $$68\%\pm 9$$ on day two, compared to $$74\%\pm 12$$ presence and $$73\%\pm 12$$ success in WG2. By approximate foraging age (day 15), SG had $$39\%\pm 8$$ presence and $$33\%\pm 7$$ success, compared to $$36\%\pm 3$$ presence and $$29\%\pm 4$$ success in WG2. The presence and success rates for the entire sampling period are in Fig. 3.The number of presence and success bees decreased as the bees increased in age (presence: Wald $$X^2$$, $$X^2=302.9$$, $$p<0.001^{***}$$, success: Wald $$X^2$$, $$X^2=331.1$$, $$p<0.001^{***}$$).

**Figure 4:**
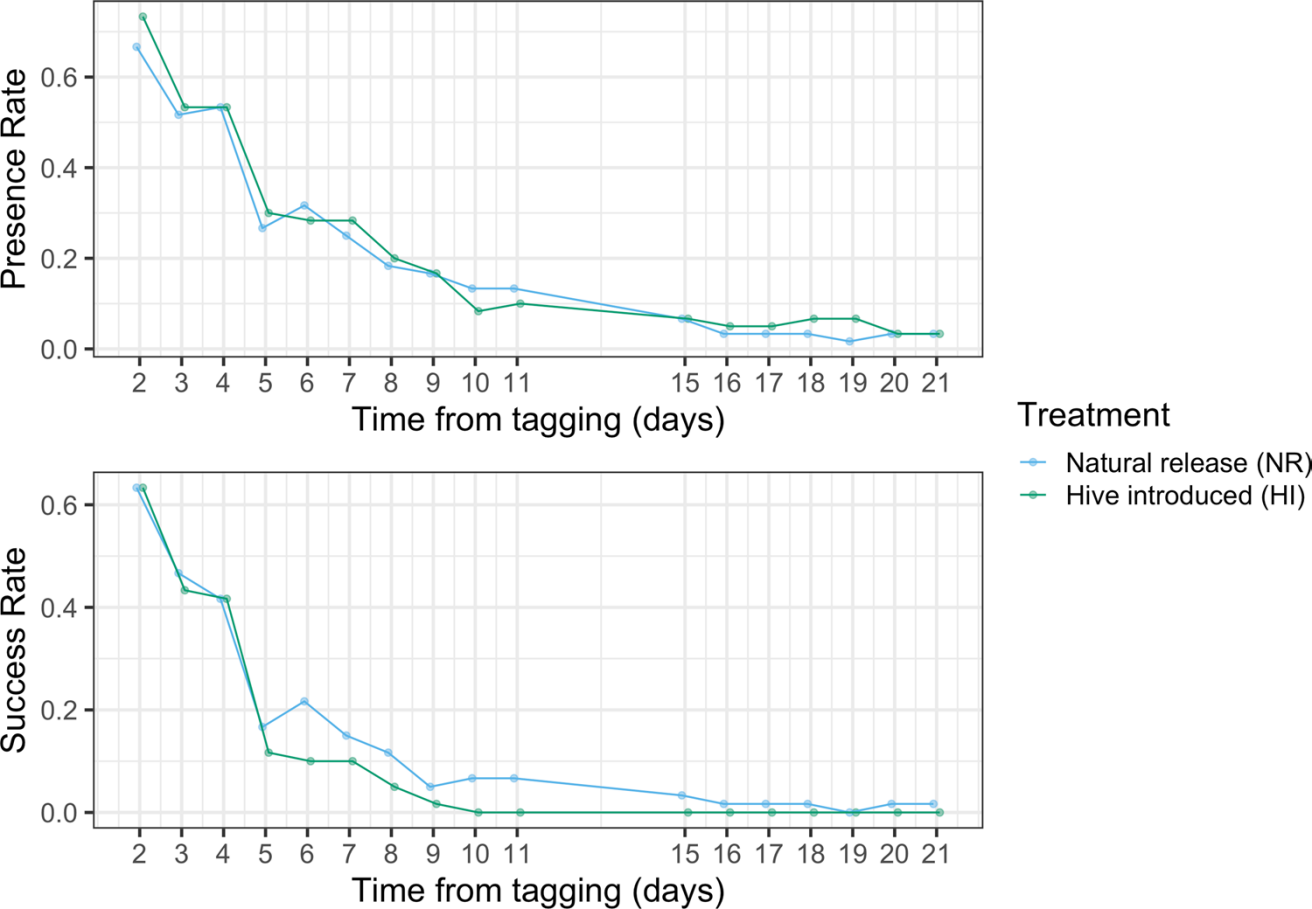
Presence and success of foragers on the days following introduction to the three observation hives. The age of foragers is unknown, as foragers were collected from hive entrances.

In experiment 3, treatment group did not significantly affect presence, but did significantly affect success (presence: Wald $$X^2$$, $$X^2=.38$$, $$p=0.54$$, success: Wald $$X^2$$, $$X^2=7.86$$, $$p=0.005^{**}$$). Natural Release foragers had slightly, but not significantly, lower presence than Hive Introduced foragers over the course of the sampling period, but had significantly higher success (presence: glmm, $$z=-0.62$$, $$p=0.54$$, success: glmm, $$z=2.80$$, $$p=0.005^{**}$$). The presence and success for HI were $$73\%\pm 6$$ and $$63\%\pm 4$$, respectively, on day 2, compared to $$67\%\pm 7$$ and $$63\%\pm 8$$ for NR. By day 15, HI had $$7\%\pm 2$$ presence and $$0\%\pm 0$$ success, compared to $$7\%\pm 2$$ presence and $$3\%\pm 3$$ success for NR. The presence and success rates for the entire sampling period are in Fig. 4. The number of presence and success bees decreased in the days following introduction, though the age of the bees was unknown since foragers were collected from the entrance of the hive (presence: Wald $$X^2$$, $$X^2=273.0$$, $$p<0.001^{***}$$, success: Wald $$X^2$$, $$X^2=202.5$$, $$p<0.001^{***}$$).

## Discussion

Introducing nurses during the night and using introduction cages were the most important techniques for increasing presence and success. Daytime and No Cage bees had significantly lower presence and success than the control group in the hive observations. This difference was not significant in the destructive sampling, but Daytime and No Cage were the groups associated with the lowest presence and success regardless of sampling method, and we would recommend that researchers introducing tagged nurse bees do so at night using an introduction cage whenever possible. Bees acquire a unique hydrocarbon profile from the comb and other workers in the colony^24^. It has been shown that honey bee colonies discriminate more against foreign smelling bees when they are likely to encounter intruders (e.g. during times when a lot of food robbing is occurring, such as forage dearths)^25^. Colonies may be more permissive at night, when intruder attempts are unlikely because foragers and guards sleep^26^. As newly introduced bees spend time in the hive, their cuticle acquires the hydrocarbon profile of the colony, and by morning they may be less likely to be recognized as an intruder. Introduction cages may increase presence and success similarly, by allowing introduced bees to acquire the colony’s hydrocarbon profile before interacting extensively with new nestmates. Additionally, rapid introduction of bees through a funnel could potentially startle the colony and provoke a defense response, leading the colony to attack newly introduced bees.

No Sucrose bees had lower presence and success compared to the control in the hive observations, but this pattern did not hold in the destructive sampling, where they had slightly, but not significantly, higher presence and success than the control group. We therefore conclude that sucrose probably does not make a big difference for presence and success of tagged bees. Since spraying sucrose on bees tagged with ASICs could damage the chip, we recommend that researchers not use sugar syrup when tagging bees with electronics.

Not all glues work well to attach tags to honey bees. Honey bees sense chemicals using their antennae, and glues often smell strongly. The bond between glue and tag can vary in strength and durability. With silicon tags, shellac glue was associated with lower presence and success than wood glue. Silicon tags attached with wood glue and plastic tags attached with shellac glue had some of the highest presence and success rates of all treatments in both the hive observations and the destructive sampling, though presence and success were only significantly higher than the control, which had silicon tags with shellac glue, in the hive observations. With silicon tags, wood glue and superglue both worked well, resulting in high success. We cannot say definitively which glue type is best because it depends on the material. Super glue and wood glue are both inexpensive and readily available glues that work with silicon tags, and are likely to work for other materials too.

When secured with shellac glue, the differences between silicon and plastic tags are small, and there were no significant differences between the Plastic and Control groups in the hive observations or destructive samplings. However, in the destructive sampling, we did find that the Plastic group had, on average, the highest success rate of any of the treatment groups. This may be because plastic queen-marking disks are rounded, allowing almost the entire surface area to contact the thorax of the bee. Additionally, flat, square tags like those we made out of silicon have corners, which may allow grooming bees more leverage when trying to remove tags.

Not Incubated bees had significantly higher presence and slightly, but not significantly lower success than control bees, which were stored in the incubator, in both the hive observations and the destructive sampling. In the destructive sampling, they had, on average, the highest presence of any group, though their success was lower than the control. Because incubators are warmer than room temperature, they may strengthen the glue bond between thorax and tag and reduce the smells associated with the glue. One possible explanation is that if tagged bees are detected and their tags are not able to be removed, they may have an increased likelihood of being ejected from the colony. If the Not Incubated bees were more easily detected by groomers than incubated bees, but their tags were more easily removed, it could explain the pattern we saw, where Not Incubated bees had higher presence but slightly lower success compared to control bees in the destructive sampling.

Overall, the presence and success rates were similar between the the Natural release and Hive introduced foragers, but we achieved significantly higher success introducing foragers with the Natural Release method, where we released them in front of their hive after tagging, than with the introduction cage method we used with nurses. This is also the least labor intensive and disruptive way to reintroduce foragers to their colony, because they are able to return to the hive by flying on their own as if returning from a foraging trip. Even when choosing healthy-appearing foragers to tag, most were missing from the hive after a few days, with less than half of those present on day 2 remaining on day 5. From this we conclude that when tagging foragers, it is important to collect as much data as possible in the first few days after tagging.

As colonies grow in size, the likelihood of seeing each bee during a hive observation decreases markedly. In our destructive sampling on day 14, we found more bees than we had seen on day two with hive observations for most treatments. When comparing results between the hive observations and destructive samplings, we found that the treatment groups that had the lowest presence and success were consistent across both sampling types, and that presence and success for the hive observations on day 9, the last observation day, were significantly correlated with presence and success in the destructive sampling on day 14. One discrepancy between these sampling methods is that we found many more Not Incubated bees present during the destructive sampling than we had previously thought, a pattern that likely occurred because the success of Not Incubated bees was so low, and high tag removal made them less visible during hive scans. Ultimately, our destructive sampling showed that 14 days after introduction, > 50% of the tagged nurse bees on average were present for all treatment groups and > 50% of bees on average were present with tags for 6 of the 7 Treatment groups. In our most successful treatment, Plastic, an average of 72% of the tagged bees were present with tags 14 days after introduction.

The best introduction method for any given project will depend on the goals of the project, equipment availability, the experience levels of taggers, and the limitations of the tag hardware. When we introduce newly hatched nurse bees to observation hives, we have the ability to track them throughout their ontogeny and know their exact ages, but tags may fall off or acquire residues before the bees reach foraging age. Residues can inhibit information gathering by covering visual tags or by preventing electronic tags from gathering or transferring data. When tagging foragers, data sampling can commence immediately, but we may get less data per tag since bees survive for an average of 7.7 days after they begin foraging and we may tag older foragers by chance^27^. The risk of tagging older foragers can be somewhat mitigated by tagging visually healthy foragers with intact wings.

Tagging nurse bees is significantly easier, faster, and safer than tagging foragers. Frames of emerging brood can be placed in an incubator overnight and all emerged bees can be brushed off in the morning. Newly hatched bees can be left in a container with petroleum jelly around the top rim, because they do not fly yet. They haven’t synthesized venom yet^28^, so they rarely sting, and when they do it’s painless (P.A.K., personal observation). They can also be collected from source colonies, as they haven’t acquired a hydrocarbon profile yet^24^. Foragers are more difficult to tag because they sting readily, so they must be anesthetized with cold or carbon dioxide. They have already acquired a hydrocarbon profile^24^, and have learned to navigate the landscape, so they must be reintroduced to their maternal colony or moved outside their foraging range. Introducing foragers works well in a time crunch, when knowing the age of foragers is not important, or when it is especially important to prevent residue build-up on tags.

## Supplementary Information

Supplementary Video 1.

## Data Availability

The datasets generated and analysed during the current study will be available in the Cornell eCommons repository upon publication at 10.7298/2by5-4g96.
